# Metabolic and Lifestyle Profiles of Metabolic Dysfunction-Associated Steatotic Liver Disease in Romanian and Italian Adults

**DOI:** 10.3390/medicina62071403

**Published:** 2026-07-20

**Authors:** Naomi-Adina Ciurea, Harshitha Shanmugam, Cristina Monica Pantea, Paul Grama, Irina-Bianca Kosovski, Simona Bataga, Agostino Di Ciaula, Piero Portincasa

**Affiliations:** 1 Department of Internal Medicine, “George Emil Palade” University of Medicine, Pharmacy, Science and Technology of Targu Mures, 540139 Targu Mures, Romaniasimonabataga@yahoo.com (S.B.); 2 Doctoral School of Medicine and Pharmacy, “George Emil Palade” University of Medicine, Pharmacy, Sciences and Technology of Targu Mures, 38 Gheorghe Marinescu Street, 540139 Targu Mures, Romania; 3 Doctoral School of Public Health, Clinical Medicine and Oncology, University of Bari “Aldo Moro”, 70121 Bari, Italy; 4 Clinica Medica “A. Murri”, Department of Precision and Regenerative Medicine and Ionian Area (DiMePre-J), University of Bari Aldo Moro, Piazza G. Cesare, 70124 Bari, Italy; harshithashanmugam993@gmail.com (H.S.); agodiciaula@gmail.com (A.D.C.); piero.portincasa@uniba.it (P.P.); 5 Department of Clinical Science-Internal Medicine, “George Emil Palade” University of Medicine, Pharmacy, Science and Technology of Targu Mures, 540139 Targu Mures, Romania; cmonicapantea@gmail.com; 6 Department of Pathophysiology, “George Emil Palade” University of Medicine, Pharmacy, Science and Technology of Targu Mures, 540139 Targu Mures, Romania

**Keywords:** MASLD, obesity, Mediterranean diet, insulin resistance, intestinal permeability, gut–liver axis, physical activity, depression, lifestyle factors

## Abstract

*Background*: Metabolic dysfunction-associated steatotic liver disease (MASLD) represents the hepatic manifestation of systemic metabolic dysfunction and has become one of the leading causes of chronic liver disease worldwide. Although obesity is a major determinant of MASLD, the metabolic and lifestyle context in which the disease develops may vary across different geographical and dietary environments. The present study aimed to evaluate the prevalence of MASLD among adults with metabolic dysfunction from two European cohorts, to compare the clinical, metabolic, lifestyle, and psychological characteristics of participants with MASLD from Romania and Italy, and to explore differences according to MASLD status within each cohort. In addition, intestinal permeability was exploratorily assessed in a subgroup of the Italian cohort. *Methods*: This prospective multicentre observational study included 132 adults undergoing metabolic and hepatic evaluation in Romania (*n* = 52) and Italy (*n* = 80). Hepatic steatosis was assessed using SteatoTest in the Romanian cohort and ultrasonography in the Italian cohort. Anthropometric and metabolic parameters were recorded in all participants. Dietary quality was evaluated using the MEDI-LITE questionnaire, physical activity using the International Physical Activity Questionnaire (IPAQ), depressive symptoms using the Patient Health Questionnaire-9 (PHQ-9), and health-related quality of life using the 36-Item Short Form Health Survey (SF-36). Intestinal permeability was evaluated in the Italian cohort using the lactulose/mannitol absorption test. *Results*: MASLD was identified in 80.8% of participants from the Romanian cohort and in 66.3% of participants from the Italian cohort. Among patients with MASLD, Italian participants exhibited significantly higher body mass index, waist circumference, fasting insulin, total cholesterol, LDL cholesterol, and diastolic blood pressure compared with Romanian participants, whereas HDL cholesterol levels were significantly higher in the Romanian cohort. Sex-stratified analyses revealed significant sex-related differences in anthropometric and metabolic parameters within both cohorts. In both geographical populations, participants with MASLD demonstrated lower adherence to the Mediterranean diet, lower physical activity levels, higher depressive symptom burden, and less favorable quality-of-life indicators compared with participants without MASLD. Exploratory analyses of intestinal permeability in the Italian cohort did not reveal significant differences according to MASLD or obesity status. *Conclusions*: MASLD was highly prevalent in both Romanian and Italian adults with metabolic dysfunction. Patients with MASLD exhibited distinct metabolic and lifestyle profiles across the two geographical cohorts, supporting the potential contribution of environmental and lifestyle-related factors to MASLD heterogeneity. Lower adherence to the Mediterranean diet, reduced physical activity, and increased psychological burden were consistently associated with MASLD in both populations. Exploratory intestinal permeability analyses did not demonstrate significant differences according to MASLD or obesity status in the Italian cohort.

## 1. Introduction

Obesity has emerged as one of the most pressing metabolic challenges of the twenty-first century. Over the past decades, the prevalence of overweight and obesity has increased dramatically worldwide, contributing to the rising burden of metabolic disorders across both developed and developing regions [1]. Excess adiposity is now recognized as a central determinant of metabolic dysfunction-associated steatotic liver disease (MASLD) [2], previously named non-alcoholic fatty liver disease (NAFLD), which has become the most common chronic liver disease globally. MASLD represents the hepatic manifestation of systemic metabolic dysfunction and is strongly associated with obesity, insulin resistance, dyslipidemia, and type 2 diabetes [3]. As the prevalence of obesity continues to rise, the global epidemiological burden of MASLD has increased in parallel, affecting approximately one third of the adult population and representing a major contributor to cardiometabolic morbidity and mortality [4]. Recent epidemiological studies indicate that MASLD affects approximately 25–35% of the adult population worldwide, with substantial regional variability across Europe. In Italy, the prevalence of MASLD has been estimated to exceed 25% in the general adult population, reflecting the increasing burden of obesity, insulin resistance, and other metabolic risk factors despite the traditional Mediterranean dietary pattern [5]. Similarly, Eastern European countries, including Romania, have experienced significant nutritional and lifestyle transitions during recent decades, contributing to a growing prevalence of obesity-related metabolic disorders and MASLD [6]. These epidemiological trends highlight the importance of evaluating MASLD within different geographical and lifestyle contexts across Europe [7].

The diagnosis of MASLD is currently based on the presence of hepatic steatosis identified by imaging, histology, or validated non-invasive tests in individuals with at least one cardiometabolic risk factor and in the absence of alternative causes of steatosis. In routine clinical practice, ultrasonography remains the most widely used first-line diagnostic tool, while elastography-based techniques and serum-based non-invasive tests may provide additional information regarding liver involvement.

Among the various histological and clinical features of MASLD, liver fibrosis is currently recognized as the strongest predictor of liver-related outcomes, cardiovascular events, and overall mortality. Consequently, early identification of patients at risk of fibrosis progression has become a major focus of contemporary MASLD management and risk stratification strategies. Nevertheless, hepatic steatosis remains the defining feature required for diagnosis and represents the earliest detectable stage of the disease spectrum.

The relationship between obesity and MASLD is complex and multifactorial [8]. Excess adipose tissue promotes hepatic fat accumulation through insulin resistance, increased free fatty acid flux to the liver, chronic low-grade inflammation, and dysregulated endocrine signaling [9]. These metabolic disturbances contribute not only to hepatic steatosis but also to progressive liver injury and fibrosis [10]. Importantly, obesity cannot be interpreted solely as an individual clinical condition but must also be understood within the broader context of environmental and societal determinants [11]. In particular, changes in dietary habits, food systems, socioeconomic inequalities, and the increasing availability of energy-dense ultra-processed foods have contributed to the emergence of obesogenic environments that promote metabolic dysfunction across many populations [12].

Within this framework, dietary patterns have received increasing attention as modifiable determinants of metabolic health [13]. The Mediterranean diet, characterized by high consumption of plant-based foods, olive oil, whole grains, legumes, fruits, vegetables, and moderate intake of fish, has consistently been associated with improved cardiometabolic profiles [14]. Numerous studies have demonstrated that adherence to the Mediterranean dietary pattern is associated with reduced obesity prevalence, improved insulin sensitivity, lower systemic inflammation, and decreased hepatic fat accumulation [15]. Beyond its nutritional composition, the Mediterranean diet has also been recognized by UNESCO as an Intangible Cultural Heritage, reflecting a broader cultural and lifestyle framework that includes traditional food preparation, seasonal eating patterns, social meals, and sustainable food practices [16].

However, dietary environments across Europe remain highly heterogeneous.

Therefore, the objectives of the present study were threefold: (1) to assess the prevalence of MASLD among adults with metabolic dysfunction from two European cohorts (Romania and Italy); (2) to compare the clinical, anthropometric, metabolic, lifestyle, and psychological characteristics of participants with MASLD across the two cohorts; and (3) to explore differences in lifestyle, psychological burden, and quality-of-life indicators according to MASLD status within each cohort. Additionally, intestinal permeability was evaluated in the Italian cohort using the lactulose/mannitol absorption test to explore its association with MASLD and obesity status.

## 2. Materials and Methods

### 2.1. Study Design and Population

This was a prospective, observational, multicenter study conducted in two tertiary referral centers in Romania and Italy. Adult participants undergoing metabolic and hepatic evaluation in outpatient clinics and day-hospital units of the participating tertiary referral centers in Romania and Italy were consecutively recruited between 2020 and 2024.

Inclusion criteria were the presence of overweight, obesity, and/or at least one metabolic dysfunction criterion.

Exclusion criteria were excessive alcohol consumption (>20 g/day for women and >30 g/day for men), viral, autoimmune, or drug-induced liver disease, decompensated cirrhosis, active malignancy, pregnancy or lactation, prior bariatric surgery, severe psychiatric disorders, and inability to provide informed consent.

A total of 132 participants were included (Romania: *n* = 52; Italy: *n* = 80).

All patients underwent assessment for liver steatosis, and a comprehensive assessment of anthropometric, metabolic and lifestyle data.

Participants from the Italian cohort (*n* = 80) also underwent exploratory assessment of intestinal permeability.

### 2.2. Ethical Approval

The study protocol was approved by the Ethics Committee of the County Clinical Emergency Hospital of Târgu Mureș, Romania (Approval No. Ad. 5004/16 February 2023), and the Ethics Committee of the Department of Medicine, University of Bari “Aldo Moro”, Italy (Study No. 65, Protocol No. 62806; FUEPEN).

All participants provided written informed consent in accordance with the Declaration of Helsinki.

### 2.3. Clinical and Anthropometric Assessment

Participants underwent standardized clinical evaluation. Measurements included body weight, height, waist and hip circumference, and blood pressure.

BMI was calculated as weight (kg)/height (m^2^) and categorized according to World Health Organization criteria with obesity defined as BMI ≥ 30 kg/m^2^. Waist circumference was measured at the midpoint between the lowest rib and the iliac crest and hip circumference at the level of the greater trochanters using a non-elastic tape. Waist-to-hip ratio was subsequently calculated.

Metabolic comorbidities, including obesity, type 2 diabetes mellitus, hypertension, and dyslipidemia, as well as relevant pharmacological treatments, were recorded based on medical history, medication use, and standard diagnostic criteria.

Smoking status and alcohol consumption were assessed through structured interviews.

Fasting blood samples were collected to determine glucose, insulin, and lipid profile parameters. Insulin resistance was estimated using the homeostasis model assessment of insulin resistance (HOMA-IR).

### 2.4. Assessment of Liver Steatosis

Hepatic steatosis was assessed using cohort-specific methods reflecting local routine clinical practice in each center. In the Romanian cohort, steatosis was evaluated using SteatoTest. In the Italian cohort it was assessed using ultrasonography (Noblus-E, Hitachi Medical, Tokyo, Japan) using a 3.5 MHz convex probe in the fasting state. Steatosis was graded semi-quantitatively based on hepatic echogenicity relative to the renal cortex (grades 0–3). Although different non-invasive diagnostic approaches were used, both methods represented the standard procedures routinely applied in the respective centers during the study period. The present study was designed to evaluate clinical, metabolic, lifestyle and psychological characteristics of participants classified as having MASLD within each cohort rather than to compare the diagnostic performance of steatosis assessment methods. For the purposes of the present analysis, MASLD was defined as the presence of hepatic steatosis identified by SteatoTest (≥S1) in the Romanian cohort and by fatty liver grade (≥1) in the Italian cohort.

Participants were subsequently stratified according to the presence or absence of MASLD for comparative analyses.

### 2.5. Dietary Assessment and Diet Quality Scoring

Adherence to the Mediterranean diet was assessed using the MEDI-LITE questionnaire, which evaluates habitual intake of nine typical Mediterranean food groups: fruits, vegetables, cereals, legumes, fish, meat and meat products, dairy products, alcohol, and olive oil [17]. Each component was scored according to consumption, with higher intake of traditional Mediterranean foods (fruits, vegetables, cereals, legumes, and fish) receiving higher scores, and higher intake of less characteristic foods (meat and dairy) receiving lower scores. Alcohol intake was categorized according to daily ethanol consumption, assigning the highest score to moderate intake. Olive oil consumption was included as an independent component reflecting its central role in Mediterranean dietary habits.

The total MEDI-LITE score ranges from 0 to 18 points, with higher scores indicating greater adherence to the Mediterranean diet [18]. Adherence was categorized as low (0–6), moderate (7–12), or high (13–18) according to established cut-offs [19]. A single scoring algorithm was applied to both sexes.

### 2.6. Assessment of Lifestyle Factors and Psychological Burden

Participants completed validated self-administered questionnaires to assess health-related quality of life, psychological well-being, and lifestyle behaviours potentially relevant to metabolic health.

Health-related quality of life was evaluated using the 36-Item Short Form Health Survey (SF-36), which assesses eight domains of physical and mental health [20]. Scores were transformed to a 0–100 scale, with higher scores indicating better perceived health status.

Depressive symptoms were screened using the Patient Health Questionnaire-9 (PHQ-9), a validated instrument assessing depressive symptom severity over the previous two weeks [21]. Total scores range from 0 to 27, with higher scores indicating greater symptom burden.

Physical activity was assessed using the International Physical Activity Questionnaire (IPAQ) and expressed as total weekly metabolic equivalent minutes (MET-min/week) [22].

All questionnaires were administered under standardized conditions by trained personnel using validated Romanian and Italian language versions when available.

### 2.7. Exploratory Assessment of Intestinal Permeability

Intestinal permeability was assessed in all participants from the Italian cohort (*n* = 80), as permeability testing was available exclusively in the Italian center as part of an ongoing investigation of gut–liver axis alterations. Consequently, permeability analyses were performed only in the Italian cohort and were not intended as direct comparisons between the Romanian and Italian cohorts.

Intestinal permeability was assessed in the Italian cohort using four orally administered saccharide probes. Stomach permeability was evaluated with 20 g of sucrose (SO), small intestine permeability with 5 g of lactulose (LA) and 1 g of mannitol (MA), and colonic permeability with 1 g of sucralose (SA), following the manufacturer’s instructions (AB Analitica s.r.l., Padova, Italy). Participants were instructed to avoid laxatives, prebiotics, probiotics, and synthetic sugars for one week and received dietary guidance to follow the day before testing. After an overnight fast, a baseline urine sample was collected, the sugar solution (250 mL in water) was ingested, and urine was collected hourly for six hours in sterile chlorhexidine-containing containers.

Urinary sugar concentrations were analysed by ultra-performance liquid chromatography coupled with tandem mass spectrometry (UPLC-MS/MS, AB Analitica, Padova, Italy). The fraction of excreted sugars was calculated relative to the amount ingested and expressed as a percentage. Deviations from expected recovery values indicated altered permeability in the stomach, small intestine, or colon. Normal values were defined as: SO < 0.15%, LA/MA ratio < 0.03, and SA < 1.5% [23].

### 2.8. Statistical Analysis

Statistical analyses were performed using NCSS v10 software (NCSS, LLC, Kaysville, UT, USA). Continuous variables were expressed as mean ± standard deviation or as median with interquartile range, as appropriate, whereas categorical variables were expressed as frequencies and percentages. Between-group comparisons were performed using Welch’s *t*-test for normally distributed continuous variables and the Mann–Whitney U test for non-normally distributed continuous variables. Categorical variables were compared using the chi-square test or Fisher’s exact test, as appropriate. Analyses were performed separately according to MASLD status and geographical cohort. Intestinal permeability analyses were performed in the Italian cohort, in which permeability testing was available for all participants. No regression or correlation analyses were performed in the present revised analytical framework.

## 3. Results

### 3.1. Study Population and Baseline Characteristics

A total of 132 participants were included in the analysis, comprising 52 patients from the Romanian cohort and 80 from the Italian cohort.

MASLD was identified in 53/80 (66.3%) participants from the Italian cohort and in 42/52 (80.8%) participants from the Romanian cohort, without statistically significant between-group difference.

Clinical and metabolic characteristics of patients with MASLD are summarized in Table 1.

Patients from the Italian cohort exhibited significantly higher BMI, waist circumference, hip circumference, fasting insulin, total cholesterol, LDL cholesterol, and diastolic blood pressure compared with Romanian participants. HDL cholesterol levels were significantly higher in the Romanian cohort. The prevalence of obesity was numerically higher among Italian patients with MASLD, although the difference did not reach statistical significance. No significant between-group differences were observed regarding age, sex distribution, waist-to-hip ratio, fasting glucose, HOMA-IR, triglycerides, or systolic blood pressure.

### 3.2. Sex-Specific Differences Among Patients with MASLD

Sex-stratified analyses revealed sex-related differences in anthropometric, metabolic, lifestyle, and psychological parameters within both geographical cohorts, as summarized in Table 2.

Sex-stratified analyses among patients with MASLD revealed significant differences in anthropometric and metabolic parameters within both geographical cohorts. Men exhibited significantly higher waist circumference, waist-to-hip ratio, triglyceride levels, and blood pressure values, whereas women demonstrated significantly higher HDL cholesterol concentrations. In the Italian cohort, women also showed slightly higher adherence to the Mediterranean diet. Psychological burden, assessed using the PHQ-9 questionnaire, tended to be higher among women, particularly in the Romanian cohort. Most SF-36 quality-of-life domains did not significantly differ according to sex within either cohort.

### 3.3. Dietary Quality, Lifestyle Factors, and Psychological Burden According to MASLD Status

Lifestyle and dietary variables were further examined according to MASLD status within each cohort. Detailed results are presented in Table 3 and Table 4.

In the Romanian cohort, patients with MASLD exhibited lower adherence to the Mediterranean diet, higher PHQ-9 scores, and lower scores across several SF-36 quality-of-life domains compared with subjects without MASLD. Participants with MASLD also tended to report lower physical activity levels and reduced perceived physical and mental well-being.

Similar trends were observed in the Italian cohort, where participants with MASLD demonstrated lower Mediterranean Diet Scores, lower physical activity levels, higher PHQ-9 scores, and less favorable quality-of-life indicators compared with subjects without MASLD. Several SF-36 domains were significantly lower among participants with MASLD, suggesting reduced perceived physical and mental well-being.

### 3.4. Intestinal Permeability in Subjects With or Without MASLD

Markers of intestinal permeability were analysed in the Italian cohort according to MASLD and obesity status. Results are summarized in Table 5 and Table 6.

No significant differences in intestinal permeability markers were observed according to MASLD status in the Italian cohort.

No significant differences in intestinal permeability markers were observed according to obesity status in the Italian cohort.

## 4. Discussion

### 4.1. Prevalence of MASLD and Geographical Differences Between Cohorts

In the present multicentre study, MASLD was highly prevalent among adults with metabolic dysfunction from both geographical cohorts, being identified in 80.8% of Romanian participants and 66.3% of Italian participants. These findings are consistent with the growing epidemiological burden of MASLD worldwide and further support the close relationship between metabolic dysfunction and hepatic steatosis [24,25]. The high prevalence observed in both cohorts likely reflects the enrichment of the study population with subjects presenting overweight, obesity, or other metabolic abnormalities.

While hepatic steatosis represents the defining diagnostic feature of MASLD, liver fibrosis is currently considered the strongest predictor of liver-related outcomes, cardiovascular events, and overall mortality. Therefore, the high prevalence of MASLD observed in both cohorts highlights the importance of early identification and risk stratification of affected individuals.

Despite similarities in overall metabolic dysfunction, important differences emerged between the Romanian and Italian populations. Among participants with MASLD, Italian patients exhibited significantly higher BMI, waist circumference, fasting insulin concentrations, total cholesterol, LDL cholesterol, and diastolic blood pressure compared with Romanian participants. In contrast, HDL cholesterol levels were significantly higher in the Romanian cohort.

These findings suggest that MASLD may develop within distinct metabolic and lifestyle environments even among European populations. The observed differences may reflect variations in dietary patterns, lifestyle behaviours, sociocultural context, and environmental exposures between the two geographical regions [3,26]. Although Italy is traditionally associated with Mediterranean dietary habits, progressive westernization of lifestyle behaviours and increasing prevalence of obesity have also been reported in Southern European countries [19]. At the same time, Eastern European populations undergoing nutritional transition may experience additional metabolic vulnerability associated with changing dietary environments and reduced adherence to traditional eating patterns [27].

Taken together, these observations support the concept that MASLD represents a heterogeneous metabolic condition whose clinical expression may vary according to geographical and environmental context [28].

### 4.2. Lifestyle Factors, Mediterranean Diet Adherence, and Psychological Burden

An important finding of the present study was the consistent association between MASLD and less favourable lifestyle-related parameters across both cohorts. In both Romanian and Italian populations, participants with MASLD demonstrated significantly lower adherence to the Mediterranean diet compared with individuals without MASLD. Lower physical activity levels and higher depressive symptom burden were also observed among participants with MASLD.

These findings are in agreement with previous evidence supporting the protective role of Mediterranean dietary patterns in metabolic and liver health [29,30]. The Mediterranean diet has been associated with improved insulin sensitivity, reduced systemic inflammation, lower hepatic fat accumulation, and better cardiovascular outcomes. Beyond nutrient composition alone, Mediterranean dietary habits also reflect broader behavioural and cultural patterns including meal structure, food quality, and lifestyle practices [31,32].

The present study further suggests that reduced physical activity and psychological burden may coexist with MASLD independently of geographical setting. Participants with MASLD from both cohorts reported lower quality-of-life scores across multiple SF-36 domains, including physical functioning, vitality, and general health perception. These observations reinforce the multidimensional nature of MASLD, extending beyond hepatic steatosis alone toward broader impairment in physical and psychological well-being [33].

The association between depressive symptoms and MASLD observed in the present analysis is biologically plausible. Chronic psychological stress and depressive burden may influence metabolic homeostasis through behavioural pathways, neuroendocrine dysregulation, altered eating behaviours, reduced physical activity, and low-grade systemic inflammation [34]. Conversely, the physical and psychosocial consequences of obesity and chronic metabolic disease may further contribute to impaired psychological health.

Together, these findings support the importance of integrated lifestyle-based approaches in MASLD management, incorporating dietary counselling, promotion of physical activity, and attention to psychological well-being [35].

### 4.3. Sex-Related Metabolic Differences in MASLD

Sex-stratified analyses revealed significant sex-related differences in anthropometric and metabolic parameters within both cohorts. Men exhibited significantly higher waist circumference and waist-to-hip ratio in both cohorts, whereas women showed significantly higher HDL cholesterol concentrations. Sex-related differences in triglyceride levels were observed only in the Romanian cohort, while higher diastolic blood pressure values were consistently observed among men in both cohorts.

These findings are consistent with previous evidence demonstrating sex-specific patterns of adipose tissue distribution and metabolic risk [36]. Visceral adiposity, more frequently observed in men, has been strongly associated with insulin resistance, systemic inflammation, and increased cardiometabolic risk [37]. By contrast, women tend to exhibit greater subcutaneous fat distribution, which may confer partial metabolic protection during earlier stages of metabolic dysfunction [38].

Interestingly, lifestyle-related variables such as physical activity and Mediterranean Diet Score showed relatively limited sex-related differences overall, suggesting that biological determinants may contribute substantially to the metabolic heterogeneity observed between sexes [39].

In the Romanian cohort, women also reported higher PHQ-9 scores compared with men, suggesting a potentially greater psychological burden among female participants with MASLD. However, most SF-36 quality-of-life domains did not significantly differ according to sex, indicating that the overall impairment in perceived health status associated with MASLD affected both sexes.

These observations further support the concept that MASLD represents a clinically heterogeneous condition influenced by complex interactions between metabolic, hormonal, behavioural, and psychosocial factors.

### 4.4. Exploratory Assessm of Intestinal Permeability

Another relevant aspect of the present study was the exploratory assessment of intestinal permeability in the Italian cohort. Because equivalent intestinal permeability testing was not available in the Romanian cohort, these analyses were not designed as direct comparisons between the two geographical populations and should be interpreted as exploratory findings. No significant differences in lactulose/mannitol ratio or prevalence of increased intestinal permeability were observed according to either MASLD or obesity status.

The relationship between intestinal permeability and MASLD has received increasing attention in recent years within the framework of the gut–liver axis [40]. Alterations in gut barrier integrity may facilitate translocation of microbial products, endotoxins, and inflammatory mediators into the portal circulation, potentially contributing to hepatic inflammation and metabolic dysregulation [41].

However, evidence regarding intestinal permeability alterations in MASLD remains heterogeneous [42]. The absence of significant differences in the present cohort may reflect several factors, including the relatively limited sample size, the exploratory nature of the analysis, or the complexity of gut barrier physiology. It is also possible that intestinal permeability alterations may be more closely associated with advanced inflammatory or fibrotic stages of liver disease rather than with MASLD presence alone [43].

Importantly, the present analytical framework intentionally restricted intestinal permeability analyses to the Italian cohort assessed using the lactulose/mannitol absorption test, avoiding direct comparison with other permeability markers measured using different methodologies.

Therefore, the current findings should be interpreted cautiously and considered exploratory. Further studies using standardized permeability assessment methods and larger populations are needed to better clarify the role of gut barrier alterations in MASLD pathophysiology.

### 4.5. Strengths and Limitations

The present study has several strengths. It included two European cohorts from distinct geographical regions and integrated metabolic, anthropometric, lifestyle, psychological, and exploratory intestinal permeability assessments within the same analytical framework. The comparative design also allowed evaluation of MASLD-related heterogeneity across different environmental and lifestyle contexts.

Several limitations should also be acknowledged. First, the sample size was relatively modest, particularly for subgroup analyses, and the findings should therefore be interpreted with appropriate caution. Consequently, the present findings should be considered exploratory and hypothesis-generating, requiring confirmation in larger multicenter studies. Second, hepatic steatosis was assessed using different non-invasive methods in the two participating centers (SteatoTest in the Romanian cohort and ultrasonographic assessment in the Italian cohort). Although both approaches are widely used in routine clinical practice, the use of different diagnostic modalities may have introduced methodological heterogeneity and should be considered when interpreting between-cohort comparisons. Third, intestinal permeability analyses were available only for the Italian cohort because these assessments were performed exclusively in one participating center; consequently, no direct comparisons with the Romanian cohort could be conducted and these findings should be regarded as exploratory. Fourth, although information regarding major metabolic comorbidities and pharmacological treatments was collected during clinical assessment, these variables were not included in the primary analytical framework of the present study and therefore were not explored in detail. It should also be noted that none of the enrolled participants were receiving GLP-1 receptor agonists during the study period. Finally, due to the observational design, causal relationships between lifestyle factors, metabolic dysfunction, and MASLD cannot be established.

## 5. Conclusions

MASLD was highly prevalent among Romanian and Italian adults with metabolic dysfunction. Although both cohorts demonstrated important metabolic impairment, patients with MASLD exhibited distinct metabolic and lifestyle profiles across the two geographical populations. Lower adherence to the Mediterranean diet, reduced physical activity, increased depressive symptom burden, and poorer quality-of-life indicators were consistently associated with MASLD in both cohorts.

These findings suggest that MASLD may represent a complex metabolic condition influenced not only by adiposity itself but also by broader lifestyle and psychosocial determinants. Further large-scale multicenter studies are needed to validate these findings and to better understand the interaction between environmental factors, behavioural patterns, and metabolic dysfunction in shaping MASLD heterogeneity across populations.

## Figures and Tables

**Table 1:** Clinical and metabolic characteristics of patients with MASLD enrolled in the Italian and Romanian cohorts. The study population was characterized by a high burden of metabolic comorbidities, including obesity, type 2 diabetes mellitus, hypertension, and dyslipidemia, consistent with the metabolic-risk profile of individuals with MASLD.

Parameter	Romania (*n* = 42)	Italy (*n* = 53)	*p*-Value
Age (years)	45.86 ± 17.15 (23.00–76.00)	48.91 ± 11.85 (20.00–70.00)	0.330
Female sex, *n* (%)	22 (52.4)	20 (37.7)	0.153
BMI (kg/m^2^)	28.85 ± 6.13 (19.37–48.45)	31.89 ± 5.68 (21.80–47.75)	0.015
Waist circumference (cm)	95.86 ± 13.87 (68.00–128.00)	100.99 ± 6.88 (80.00–129.00)	0.032
Hip circumference (cm)	96.79 ± 15.62 (66.00–127.00)	107.04 ± 6.18 (90.00–130.00)	<0.001
Waist-to-hip ratio	0.96 ± 0.06 (0.85–1.19)	0.94 ± 0.06 (0.78–1.20)	0.106
Obesity (BMI ≥ 30 kg/m^2^), *n* (%)	17 (40.5)	32 (60.4)	0.054
Fasting glucose (mg/dL)	96.74 ± 20.81 (62.00–209.00)	90.55 ± 14.23 (71.00–144.00)	0.104
Fasting insulin (µU/mL)	11.21 ± 4.96 (4.00–26.00)	15.25 ± 9.11 (4.30–47.90)	0.007
HOMA-IR	2.78 ± 1.79 (0.80–11.00)	3.59 ± 2.59 (0.76–14.78)	0.079
Total cholesterol (mg/dL)	169.31 ± 38.32 (103.00–248.00)	191.47 ± 37.66 (112.00–270.00)	0.006
HDL cholesterol (mg/dL)	57.48 ± 11.69 (37.00–89.00)	49.85 ± 13.18 (26.00–89.00)	0.004
LDL cholesterol (mg/dL)	79.25 ± 23.19 (33.00–155.00)	113.80 ± 34.07 (40.60–173.20)	<0.001
Triglycerides (mg/dL)	142.80 ± 63.55 (50.00–409.00)	139.09 ± 112.72 (45.00–612.00)	0.840
Systolic blood pressure (mmHg)	129.67 ± 8.84 (115.00–151.00)	127.25 ± 10.87 (105.00–156.00)	0.248
Diastolic blood pressure (mmHg)	71.57 ± 8.01 (57.00–89.00)	79.45 ± 5.74 (69.00–94.00)	<0.001

Values are presented as mean ± standard deviation (min–max) or number (percentage), as appropriate. Continuous variables were compared using Welch’s *t*-test, and categorical variables were analysed using the Chi-square test (two-sided α = 0.05). BMI, body mass index; MASLD, metabolic dysfunction-associated steatotic liver disease; HOMA-IR, homeostasis model assessment of insulin resistance.

**Table 2:** Sex-specific differences in metabolic, clinical, and lifestyle parameters within the Romanian and Italian cohorts of patients with MASLD.

Variable	Romania Women (*n* = 22)	Romania Men (*n* = 20)	*p*-Value	Italy Women (*n* = 20)	Italy Men (*n* = 33)	*p*-Value
Age (years)	47.3 ± 16.8	44.2 ± 17.7	0.562	50.4 ± 10.7	48.0 ± 12.5	0.478
BMI (kg/m^2^)	29.6 ± 6.9	28.0 ± 5.2	0.391	31.0 ± 5.1	32.4 ± 6.0	0.372
Waist circumference (cm)	89.8 ± 11.8	102.5 ± 11.3	<0.001	94.6 ± 6.9	104.8 ± 4.7	<0.001
Hip circumference (cm)	103.8 ± 14.1	89.1 ± 10.2	<0.001	110.1 ± 5.9	105.2 ± 5.7	0.006
Waist-to-hip ratio	0.86 ± 0.04	1.04 ± 0.05	<0.001	0.86 ± 0.05	0.99 ± 0.05	<0.001
Fasting glucose (mg/dL)	93.2 ± 17.5	100.6 ± 23.7	0.245	88.1 ± 13.0	92.0 ± 15.0	0.336
Fasting insulin (µU/mL)	10.7 ± 5.0	11.8 ± 4.9	0.471	14.1 ± 8.0	15.9 ± 9.8	0.488
HOMA-IR	2.5 ± 1.5	3.1 ± 2.0	0.254	3.2 ± 2.0	3.8 ± 2.9	0.401
Total cholesterol (mg/dL)	177.5 ± 38.7	160.3 ± 36.3	0.145	198.9 ± 38.0	187.1 ± 37.1	0.283
HDL cholesterol (mg/dL)	63.2 ± 10.7	51.1 ± 9.4	<0.001	55.7 ± 13.6	46.3 ± 11.9	0.012
LDL cholesterol (mg/dL)	81.7 ± 24.5	76.5 ± 21.8	0.464	117.9 ± 35.2	111.3 ± 33.5	0.497
Triglycerides (mg/dL)	120.1 ± 46.2	167.7 ± 71.4	0.014	126.7 ± 74.8	146.6 ± 129.8	0.507
Systolic blood pressure (mmHg)	126.0 ± 7.8	133.8 ± 8.4	0.003	123.6 ± 9.8	129.5 ± 10.9	0.055
Diastolic blood pressure (mmHg)	68.7 ± 7.5	74.8 ± 7.2	0.011	76.8 ± 5.2	81.0 ± 5.5	0.009
Mediterranean Diet Score	9.8 ± 1.9	8.6 ± 2.1	0.061	11.8 ± 1.7	10.7 ± 2.0	0.047
Physical activity (MET-min/week)	1710 ± 820	2140 ± 990	0.132	2410 ± 1100	2660 ± 1240	0.456
PHQ-9 score	8.4 ± 4.1	6.0 ± 3.5	0.049	7.2 ± 3.9	5.8 ± 3.4	0.176
Physical functioning (SF-36)	67.2 ± 18.1	72.9 ± 17.0	0.301	74.7 ± 16.2	76.9 ± 15.7	0.628
Role limitation—physical (SF-36)	58.6 ± 20.4	65.8 ± 18.8	0.242	70.8 ± 19.1	72.1 ± 17.7	0.807
Bodily pain (SF-36)	60.9 ± 19.5	68.0 ± 17.4	0.214	69.7 ± 18.3	71.2 ± 17.9	0.769
General health (SF-36)	56.2 ± 16.9	61.0 ± 15.2	0.336	65.8 ± 15.8	66.5 ± 15.2	0.872
Energy/Vitality (SF-36)	48.7 ± 15.4	56.4 ± 14.7	0.101	62.1 ± 14.9	65.0 ± 13.7	0.474
Social functioning (SF-36)	66.8 ± 18.7	72.2 ± 17.1	0.332	75.6 ± 17.4	77.5 ± 16.2	0.694
Role limitation—mental (SF-36)	60.1 ± 19.8	64.9 ± 18.2	0.418	71.5 ± 18.0	72.8 ± 17.3	0.801
Mental health (SF-36)	57.9 ± 15.6	64.2 ± 14.7	0.185	69.5 ± 14.1	71.8 ± 13.5	0.556

Values are expressed as mean ± standard deviation. *p*-values represent comparisons between women and men within each cohort. Comparisons were performed using independent samples *t*-test or Mann–Whitney U test, as appropriate. BMI, body mass index; HOMA-IR, homeostatic model assessment of insulin resistance; MET, metabolic equivalent of task; PHQ-9, Patient Health Questionnaire-9; SF-36, Short Form Health Survey-36.

**Table 3:** Dietary quality, physical activity, and psychological burden according to MASLD status in the Romanian cohort.

Parameter	With MASLD (*n* = 42)	Without MASLD (*n* = 10)	*p*-Value
Mediterranean Diet Score	9.1 ± 2.1	10.8 ± 1.9	0.028
Physical activity (MET-min/week)	1890 ± 940	2480 ± 1120	0.094
PHQ-9 score	7.2 ± 3.9	4.8 ± 2.7	0.041
Physical functioning (SF-36)	70.1 ± 17.8	80.6 ± 15.4	0.083
Role limitation—physical (SF-36)	62.0 ± 19.7	76.4 ± 16.2	0.037
Bodily pain (SF-36)	64.3 ± 18.6	76.8 ± 15.7	0.049
General health (SF-36)	58.5 ± 16.2	71.0 ± 14.8	0.031
Energy/Vitality (SF-36)	52.4 ± 15.2	67.8 ± 14.0	0.008
Social functioning (SF-36)	69.4 ± 18.0	79.9 ± 16.3	0.091
Role limitation—mental (SF-36)	62.3 ± 19.1	75.2 ± 16.8	0.052
Mental health (SF-36)	60.8 ± 15.1	72.5 ± 13.7	0.024

Values are presented as mean ± standard deviation. Continuous variables were compared using the Mann–Whitney U test (two-sided α = 0.05). Physical activity was expressed as total weekly metabolic equivalent minutes (MET-min/week). The PHQ-9 score reflects depressive symptom burden and does not represent a clinical psychiatric diagnosis. SF-36 domain scores range from 0 to 100, with higher scores indicating better perceived health status.

**Table 4:** Dietary quality, physical activity, and psychological burden according to MASLD status in the Italian cohort.

Parameter	With MASLD (*n* = 53)	Without MASLD (*n* = 27)	*p*-Value
Mediterranean Diet Score	11.1 ± 1.9	12.4 ± 1.6	0.012
Physical activity (MET-min/week)	2570 ± 1190	3180 ± 1280	0.048
PHQ-9 score	6.3 ± 3.7	4.2 ± 2.9	0.029
Physical functioning (SF-36)	76.1 ± 15.8	84.9 ± 13.6	0.022
Role limitation—physical (SF-36)	71.6 ± 18.1	81.2 ± 15.5	0.031
Bodily pain (SF-36)	70.6 ± 18.0	79.5 ± 14.8	0.046
General health (SF-36)	66.2 ± 15.4	75.7 ± 13.9	0.019
Energy/Vitality (SF-36)	63.9 ± 14.2	74.3 ± 12.8	0.008
Social functioning (SF-36)	76.8 ± 16.7	84.2 ± 14.5	0.071
Role limitation—mental (SF-36)	72.3 ± 17.5	80.6 ± 15.3	0.058
Mental health (SF-36)	70.9 ± 13.8	78.4 ± 12.6	0.024

Values are presented as mean ± standard deviation. Continuous variables were compared using Welch’s *t*-test (two-sided α = 0.05). Physical activity was expressed as total weekly metabolic equivalent minutes (MET-min/week). The PHQ-9 score reflects depressive symptom burden and does not represent a clinical psychiatric diagnosis. SF-36 domain scores range from 0 to 100, with higher scores indicating better perceived health status. No correction for multiple comparisons was applied due to the exploratory nature of the analysis.

**Table 5:** Markers of intestinal permeability according to MASLD status in the Italian cohort.

Variable	With MASLD (*n* = 53)	Without MASLD (*n* = 27)	*p*-Value
LA/MA ratio	0.01 [0.01–0.02]	0.01 [0.01–0.02]	0.512
Increased intestinal permeability (LA/MA > 0.03), *n* (%)	4 (7.5)	1 (3.7)	0.648

Values are presented as median [interquartile range] or number (percentage), as appropriate. Between-group comparisons were performed using the Mann–Whitney U test for continuous variables and Fisher’s exact test for categorical variables (two-sided α = 0.05). Intestinal permeability was assessed using the lactulose/mannitol absorption test in the Italian cohort.

**Table 6:** Markers of intestinal permeability according to obesity status among Italian participants (*n* = 80; non-obese *n* = 48, obese *n* = 32).

Variable	Non-Obese (BMI < 30 kg/m^2^)	Obese (BMI ≥ 30 kg/m^2^)	*p*-Value
LA/MA ratio	0.01 [0.01–0.02]	0.01 [0.01–0.02]	0.426
Increased intestinal permeability (LA/MA > 0.03), *n* (%)	3 (6.3%)	2 (6.3%)	1.000

Values are presented as median [interquartile range] or number (percentage), as appropriate. Obesity was defined as body mass index (BMI) ≥ 30 kg/m^2^. Between-group comparisons were performed using the Mann–Whitney U test for continuous variables and Fisher’s exact test for categorical variables (two-sided α = 0.05). Intestinal permeability was assessed using the lactulose/mannitol absorption test in the Italian cohort.

## Data Availability

The data presented in this study are available upon request from the corresponding author.

## Abbreviations

The following abbreviations are used in this manuscript:

BMI	Body Mass Index
HDL	High-Density Lipoprotein
HOMA-IR	Homeostasis Model Assessment of Insulin Resistance
IPAQ	International Physical Activity Questionnaire
LA	Lactulose
LA/MA	Lactulose–Mannitol Ratio
LDL	Low-Density Lipoprotein
MA	Mannitol
MASLD	Metabolic Dysfunction-Associated Steatotic Liver Disease
MEDI-LITE	Mediterranean Diet Literature-Based Adherence Score
MET Metabolic	Equivalent of Task
MET-min/week	Metabolic Equivalent Task Minutes per Week
NCSS	Number Cruncher Statistical System
PHQ-9	Patient Health Questionnaire-9
SA	Sucralose
SF-36	36-Item Short Form Health Survey
SO	Sucrose
UPLC-MS/MS	Ultra-Performance Liquid Chromatography Coupled with Tandem Mass Spectrometry
